# Acclimatizing and training freely viewing marmosets for behavioral and electrophysiological experiments in oculomotor tasks

**DOI:** 10.14814/phy2.15594

**Published:** 2023-02-08

**Authors:** Seyed Javad Saghravanian, Ali Asadollahi

**Affiliations:** ^1^ Visuo‐Motor Systems Laboratory, Department of Biology Ferdowsi University of Mashhad Mashhad Iran; ^2^ Present address: Washington National Primate Research Center, and Department of Biological Structures University of Washington Seattle WA USA

**Keywords:** eye tracking, marmoset, oculomotor, superior colliculus

## Abstract

The marmoset is a small‐bodied primate with behavioral capacities and brain structures comparable to macaque monkeys and humans. Its amenability to modern biotechnological techniques like optogenetics, chemogenetics, and generation of transgenic primates have attracted neuroscientists' attention to use it as a model in neuroscience. In the past decade, several laboratories have been developing and refining tools and techniques for performing behavioral and electrophysiological experiments in this new model. In this regard, we developed a protocol to acclimate the marmoset to sit calmly in a primate chair; a method to calibrate the eye‐tracking system while marmosets were freely viewing the screen; and a procedure to map motor field of neurons in the SC in freely viewing marmosets. Using a squeeze‐walled transfer box, the animals were acclimatized, and chair trained in less than 4 weeks, much shorter than what other studies reported. Using salient stimuli allowed quick and accurate calibration of the eye‐tracking system in untrained freely viewing marmosets. Applying reverse correlation to spiking activity and saccadic eye movements, we were able to map motor field of SC neurons in freely viewing marmosets. These refinements shortened the acclimation period, most likely reduced stress to the subjects, and allowed more efficient eye calibration and motor field mapping in freely viewing marmosets. With a penetration angle of 38 degrees, all 16 channels of the electrode array, that is, all recorded neurons across SC layers, had overlapping visual receptive and motor fields, indicating perpendicular penetration to the SC.

## Graphical abstract

This study describes refinements of chair acclimation, eye calibration and motor field mapping of SC neurons that shortened the acclimation period, and allowed more efficient eye calibration and motor field mapping in freely viewing marmosets.

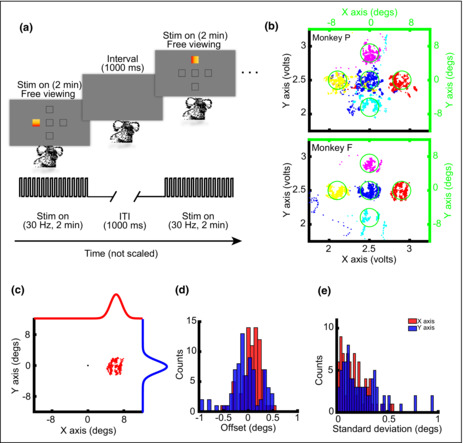

## 1 INTRODUCTION

In the past 50 years, due to its ability to perform complex cognitive tasks and its behavioral and brain similarities to humans, the rhesus macaque has been the most common primate model for oculomotor and visual neuroscience investigations (Basso & May, 2017; Goldberg & Wurtz, 1972; Krauzlis et al., 2013; Wurtz, 1969b). The temporal and spatial resolution of invasive neurophysiological techniques in behaving macaques has provided an incomparable preparation for mapping the motor and cognitive systems. However, the use of modern molecular and imaging techniques is very limited in the macaque monkey. These techniques are necessary for analyzing cognitive systems at the mechanistic and cellular levels and have been well established and vastly used in laboratory rodents. Despite their amenability to biotechnological tools, rodents have limited capacity to perform complex cognitive and motor tasks, and their oculomotor and visual systems differ in many ways from primates, making it difficult to translate discoveries in rodents to non‐human primates and humans (D'Souza et al., 2021).

The common marmoset is a small new‐world monkey (adults weigh around 400 g) amenable to biotechnological techniques. Investigators have been able to create genetically modified marmosets (Kishi et al., 2014; Sasaki, 2015; Sasaki et al., 2009; Shen, 2013; Tomioka et al., 2017), manipulate neuronal circuits using optogenetics (Ebina et al., 2019; Macdougall et al., 2016) and chemogenetics (Mimura et al., 2021), and image the activity of populations of neurons in the marmoset (Ebina et al., 2018; Yamada et al., 2016); hence, it potentially can bridge the gap between neurobiological findings in the rodents and the macaque monkey (D'Souza et al., 2021; Mitchell et al., 2014).

The marmoset oculomotor system shares many behavioral and neural activity characteristics with the macaque monkey and people (Chen et al., 2021; Ghahremani et al., 2017; Johnston et al., 2018; Mitchell et al., 2014; Mitchell & Leopold, 2015; Solomon & Rosa, 2014; Spadacenta et al., 2019). The saccadic kinematics, for saccades up to 25° in amplitude, is similar in these three species (Chen et al., 2021). Use of the marmoset in neuroscience of oculomotor function can be complementary to the finding of the macaque monkey studies in several ways. The marmoset lessencephalic brain allows imaging, array‐based electrophysiology and optogenetics in two important cortical oculomotor areas that are buried in the sulci in the macaque monkey, the frontal eye field (FEF), and the lateral intraparietal areas (LIP) (Ghahremani et al., 2019; Johnston et al., 2019; Schaeffer et al., 2019; Selvanayagam et al., 2019). Although oculomotor and perceptual function of the midbrain oculomotor hub, the superior colliculus, is known to a great extent, function of different cell types in the SC, and their interaction with the other sensory‐motor midbrain nuclei, like the PPT or the PBG, is less known in primates. Use of highly desired histological and cytological tools as well as neurophysiological experiments in the marmoset can complement macaque studies, and result in understanding SC function at the cellular and mechanistic levels. This advantage is due to better availability of the marmoset relative to the macaque monkey, primarily due to the smaller size of the marmoset, and its amenability to biotechnological tools. We recorded activity of SC neurons using linear arrays in behaving marmosets, and mapped their visual receptive field and motor fields, prerequisite steps for studying the marmoset midbrain oculomotor system.

Acclimatizing marmosets to sit calmly in a primate chair under head‐restrained condition is fundamental to many oculomotor and vision studies. Although these techniques have increasingly been used in the past decade, it takes a considerably long time to make an animal acclimatized, and ready for oculomotor experiments (2–3 months in Nummela et al. (2017), 6–8 weeks in Johnston et al. (2018), more than 200 days in Sedaghat‐Nejad et al. (2019)). Maybe originally adopted from rodent handling procedures, investigators use a plexiglass tube to transfer marmosets from a transfer box to a chair, a protocol that requires handling and touching the animals (Lu et al., 2001; Remington et al., 2012). The marmoset is a small prey species and dislikes being touched by an experimenter, so it takes a long time for them to get used to it. Here, we describe a squeeze‐walled plexiglass transfer box that helps experimenters to avoid touching the animals, thus shortening the acclimation period.

The marmoset, like human and other non‐human primates, constantly orients its gaze and attention to the location of important objects by saccadic eye movements. Eye tracking is used to study eye movements in oculomotor and visual tasks or to have the animal fixate the monitor center for presenting a visual stimulus on a specific location of the visual field relative to the foveal representation. Calibration of the eye tracker system is fundamental to accurate eye tacking and is a challenging step in an untrained marmoset. Some investigators used a salient stimulus like a marmoset face (Ghahremani et al., 2019; Mitchell et al., 2014; Nummela et al., 2017; Selvanayagam et al., 2019), colored spots, or moving pictures (Spadacenta et al., 2019) to make the subject fixate for a short period, while they manually captured fixations (e.g., by a key press), a frustrating process in a naïve marmoset (16, 30). In all these protocols, the animal actively fixates the stimuli, and it is rewarded for long‐enough fixations. This is a challenging procedure in a naïve animal, and even in a trained marmoset, it might take a full experimental session to calibrate the eye tracking system. In a refined protocol, we calibrated the eye tracking system by displaying salient stimuli and recording eye signals of untrained freely viewing marmosets. This refined calibration protocol in freely viewing marmosets is quick, accurate, and doable in untrained marmosets without rewarding them for fixations.

Refinement of basic electrophysiological procedures like receptive field and motor field mapping, which are necessary steps in most oculomotor experiments, is another strategy to save time and trials for the main cognitive task. Traditionally, neural responses to stimuli with various parameters of a feature, that is, location or orientation, are measured, or neural responses are cross‐correlated to randomly presented brief stimuli to map receptive field of neuros in various brain areas (Jendritza et al., 2021; Ringach, 2004). To characterize the motor field of the neurons in oculomotor structures, researchers use electrical macrostimulation or visually guided saccade (VGS) or memory‐guided saccade (MGS) tasks, procedures that the subjects are rewarded for actively performing the task (Massot et al., 2019). To characterize the motor field of the SC neurons, instead of using VGS, MGS or microstimulation, we correlated spiking activity of a neuron to the amplitude and direction of saccadic eye movements while the subject freely viewed the screen. This protocol does not require the marmoset to perform the tasks; therefore, it saves trials for the main oculomotor task.

Overall, reported improvements shortened the time required to acclimate marmosets for electrophysiological and behavioral experiments. Moreover, it resulted in more efficient completion of basic procedures like eye calibration, receptive field and motor field mapping in each experimental session, saving time and trials for the main cognitive task.

## 2 MATERIALS AND METHODS

### 2.1 Animal preparation and housing

Two common marmosets (*Callithrix jacchus*; subject F was male 3 years old weighing 450 g; subject R was female 7 years old weighing 370 g) and one male black‐tufted marmoset (*Callithrix penicillate*; subject P was 5 years old weighing 370 g) participated in this study. Only animals F and P were used for new eye calibration and electrophysiological recordings. Animals were housed in the Ferdowsi University of Mashhad animal facility under a 12/12 light–dark cycle, 25 ± 4°C temperature, and 40%–70% humidity. They were fed twice a day with primate food, fruits, nuts, superworms, and acacia gum and were under UV‐B light for 1.5 h once a week. Under this condition, the animals in our colony were healthy; a breeding pair thrived to ten in 30 months. The animals had ad libitum access to food and water throughout this study. All procedures were performed following the National Institutes of Health's Guide for the Care and Use of Laboratory Animals and a protocol approved by the Ethics Committee for Animal Care and Use of the Ferdowsi University of Mashhad with the code number of IR.UM.REC.1399.145.

### 2.2 Chair training using squeeze‐walled transfer boxes

Training marmosets to comfortably sit in their chair under head‐restrained condition is necessary to carry out behavioral and neurophysiological experiments in oculomotor tasks, and the procedure usually takes up to 2–3 months (Sedaghat‐Nejad et al., 2019, longer than 3 months; Johnston et al., 2018, 2 months; Nummela et al., 2017, 2–3 months). All past studies share a critical component in handling these prey species that the animals dislike, and might be the reason for lengthy training procedures, that is, touching them by hand. We designed a squeeze‐walled plexiglass transfer box and developed a protocol that guided marmosets to their chair tube without needing the experimenter to touch the subjects directly.

**FIGURE 1:**
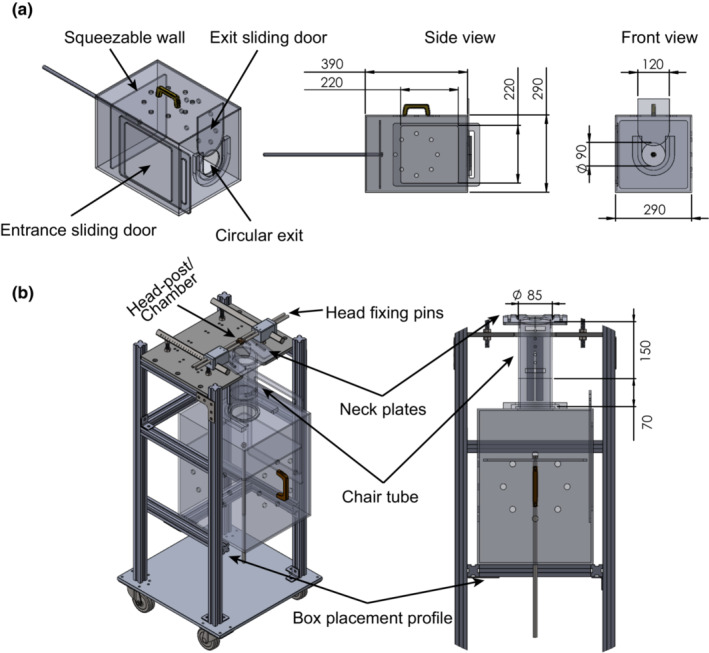
Schematic illustration of marmoset box with squeezable wall. (a) 3D view shows the entrance opening, the exit opening, and the squeezable wall. (b) The box is placed underneath the chair tube on an aluminum profile to chair the animal. Dimensions are in mm.

The transfer box (390 × 290 × 290 mm, Figure 1a) had a square‐shaped entrance (220 × 220 mm) on one side and a circular exit opening (90 mm in diameter) on the frontal side. A squeezable wall in the back of the box, opposite the exit opening, could move towards the exit opening and guide the animal to the chair tube. The chair tube was a plexiglass tube restraining the body, with two hemi‐circular neck plates at the top to restrain the neck and a baseplate at the bottom to support the legs. The chair tube was installed on an aluminum frame (Johnston et al., 2018; O'byrne & Morris, 1988; Remington et al., 2012).

To load a marmoset into its chair, the transfer box was placed on an aluminum fixture attached to the side of the cage opposite a sliding rectangular door. After a few acclimation sessions, the animal was immediately moved into the box to receive a palatable treat like a mealworm or a piece of marshmallow. Then, the box was positioned on an aluminum profile underneath the chair tube, with the box's circular opening contacting the lower edge of the chair tube (Figure 1b). The exit door was opened, the squeeze wall was slowly squeezed, and the neck plates were tightened to restrain the neck as the animal climbed into its chair tube. The entire process of training a naive marmoset to sit in the chair took less than a week. After the animals comfortably sat in their chair, head restraining began by holding their implant (see below for head‐post implant procedure) for several seconds while offering them palatable treats. After a few sessions, the head was stabilized to the stereotaxic frame using 4 fixating pins. The entire process took less than 3 weeks.

### 2.3 Head‐post/chamber implantation surgery

Before chair training, we installed a variant of the head‐post/chamber developed by Johnston et al. (2018). The inside‐hollow design of the structure allowed access to the brain for recording. Four conical receptacles on the outer sides at the anterior and posterior extent of the chamber allowed stabilizing the implant by four movable male fixating pins attached to a stereotaxic frame. The chamber was custom designed, made of titanium, and it was smaller (21.5 × 17.5 mm) than the chamber used by Johnston et al. (2018). Ten titanium screws (sizes 1.5 × 6 mm) and bone cement (Synicem, Synimed) were used to anchor the head‐post to the skull.

Twelve hours before the surgery, food was removed from the cage, and the animals had access to water only. Surgery was performed under sterile conditions and general isoflurane‐induced anesthesia. In our first monkey, monkey R, we induced anesthesia with a single dose of ketamine (15 mg/kg). Later, the monkeys P and F were placed in a plexiglass isoflurane chamber (250 × 250 × 400 mm) connected to an anesthesia machine to induce anesthesia. To maintain anesthesia, isoflurane (2%) was delivered using a custom‐made mask attached to the palate bar of the stereotaxic instrument. Temperature, heart rate, and blood oxygenation levels were monitored throughout the surgery, and the temperature was maintained using a heat blanket. After positioning the animal in the stereotaxic instrument, the hair was removed and the scalp was prepared with three alternating scrubs of ethanol (75%) and betadine (10%). A rostrocaudal incision was made, muscles were moved laterally, and the skull surface was cleaned and dried. Ten small titanium screws were bolted into the skull. The head‐post/chamber was positioned on the skull stereotaxically and anchored to the screws with acrylic‐based bone cement. Bone cement was added in very thin layers to avoid overheating. The procedure usually took 4–5 h, and the animal returned to an individual clean cage after receiving a single dose of morphine (1 mg/kg; IM). NSAID meloxicam (0.2 mg/kg on the first day and 0.1 mg/kg on the second day; SC) was administered to relieve pain and inflammation. At the beginning of the surgery, the antibiotic cefazolin (25 mg/kg every 90 min) was administered intramuscularly, and the treatment continued for 5 days (25 mg/kg; every 12 h).

To access the SC for electrophysiological recordings, the craniotomy location was planned and the electrode angle was determined based on structural MRI scans (1.5 T, Qaem Hospital Imaging Center, Mashhad, Iran). In a second surgery, a burr hole (4 × 3 mm size, 1 mm lateral to the midline and angled 38 degrees posteriorly) opened to access the brain for the SC recording.

### 2.4 Eye calibration and behavioral training of head‐fixed marmosets

We started behavioral training of the animals after a 4–6 weeks recovery period and 2–4 weeks of acclimation to the chair under head‐restrained conditions. The behavioral task was controlled by a computer running MonkeyLogic toolbox (NIMH MonkeyLogic 2), a MATLAB (The MathWorks Inc.) based toolbox available on https://monkeylogic.nimh.nih.gov/. Visual stimuli presented on a LED monitor (ViewSonic 27″, Model VX2776‐smhd) at a screen resolution of 1920 × 1080 pixels (60 Hz, 8‐bit color depth) positioned at 54 cm distance, resulted in a viewing angle of 56 degrees horizontally and 31 degrees vertically. Stimulus presentation time was recorded using a photodiode placed at the left lower corner of the screen, and it was hidden by black tape. The photodiode signal defined the onset of the stimulus presentation on the screen and was synchronously recorded with the eye and neural signal using the same data acquisition system. The eye direction was recorded using an eye‐tracking system (Bina; ToosBioResearch) with 120 frames per second.

**FIGURE 2:**
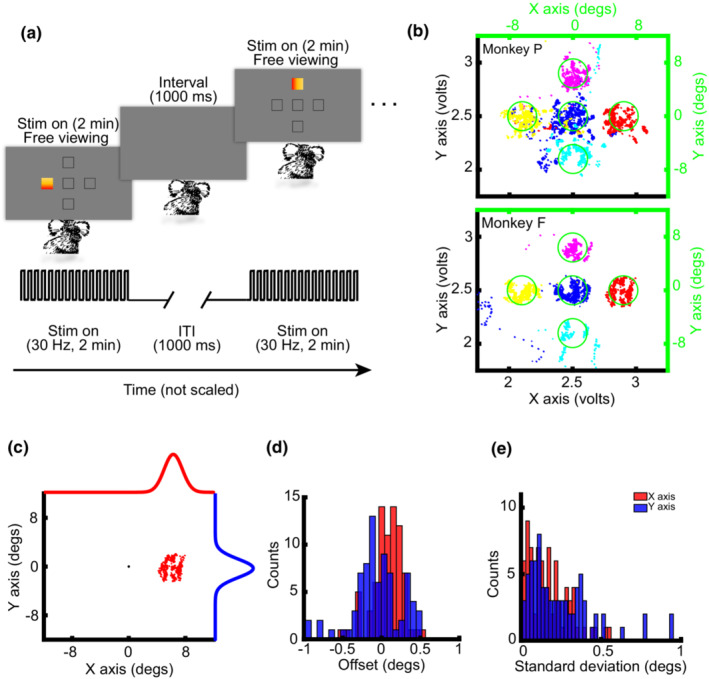
Eye calibration task and analysis. (a) Schematic representation of the calibration task. A flickering stimulus presented at 30 Hz for 2 min at each of five locations. (b) Eye tracker output during fixations of longer than 500 ms on flickering‐colored stimuli according to the raw output voltage of the eye‐tracking system (down and left black axes) and position of the stimuli on the screen (green circles according to the up and right green axes; in degrees) for monkey P and F (each color represents the fixations during presentation of a visual stimulus at a specific location). (c) A Gaussian function fitted to the distribution of fixations in *X* (red) and *Y* (blue) axes after calibration. (d) Histogram of offsets of *X* (red) and *Y* (blue) eye position during the fixations in 76 experimental sessions. The offsets calculate based on the average difference between fixation locations and the stimulus locations. (e) Histograms of standard deviation of the fixation offsets during the same 76 sessions in panel D for *X* (red) and *Y* (blue) axes.

Although it is an essential step for eye tracking, calibration is a challenge in untrained marmosets. To overcome this challenge, we used visually salient stimuli on which the marmosets spontaneously fixate. The stimuli were one degree flickering‐colored spots (flickered at 30 Hz) presented at one of five locations ([0,0], [7,0], [0,7], [−7 0], [0–7]) for 2 min at each location (Figure 2a). The subject freely viewed the screen, and it was randomly rewarded to remain alert and engaged. So, calibration was doable in an untrained animal and task completion did not depend on volunteer fixations during these 2 min periods. The eye tracker's voltage traces for the *X* and *Y* axes were filtered (20 ms moving average window), and periods of stable voltages in both *X* and *Y* axes for longer than 500 ms were considered as fixations. Fixations were visualized in a Cartesian coordinate system, resulted in five conspicuous clusters (different colors in Figure 2b). Locations of displayed stimuli were plotted (in degrees) on the same plot as the fixations were plotted (Figure 2b, green circles). A Gaussian function (formula 1) fitted to the distribution of fixations for each axis, and the center and standard deviation were extracted from the fit (Figure 2c).

Formula (1):

$$f\left(x\right)=a\times e^{-{\left(\frac{x-b}{c}\right)}^{2}}$$

where *a* is the amplitude, *b* is the centroid (location), and *c* is related to the distribution width.

The extracted Gaussian centers in the *X* and *Y* axes were used to set the gain and to remove the offset in the corresponding *X* and *Y* axes on the eye tracking system. We calibrated the system using this protocol in the first experimental session. This entire calibration process took less than 15 min. In later sessions, we manually removed the offset in the *X* and *Y* axes based on the distance between fixations and the fixation stimulus (Fixation Point; FP). The histograms of the difference (averages across many trials in a session) between stimulus location and eye direction after calibration are shown for 76 experimental sessions (Figure 2d,e).

**FIGURE 3:**
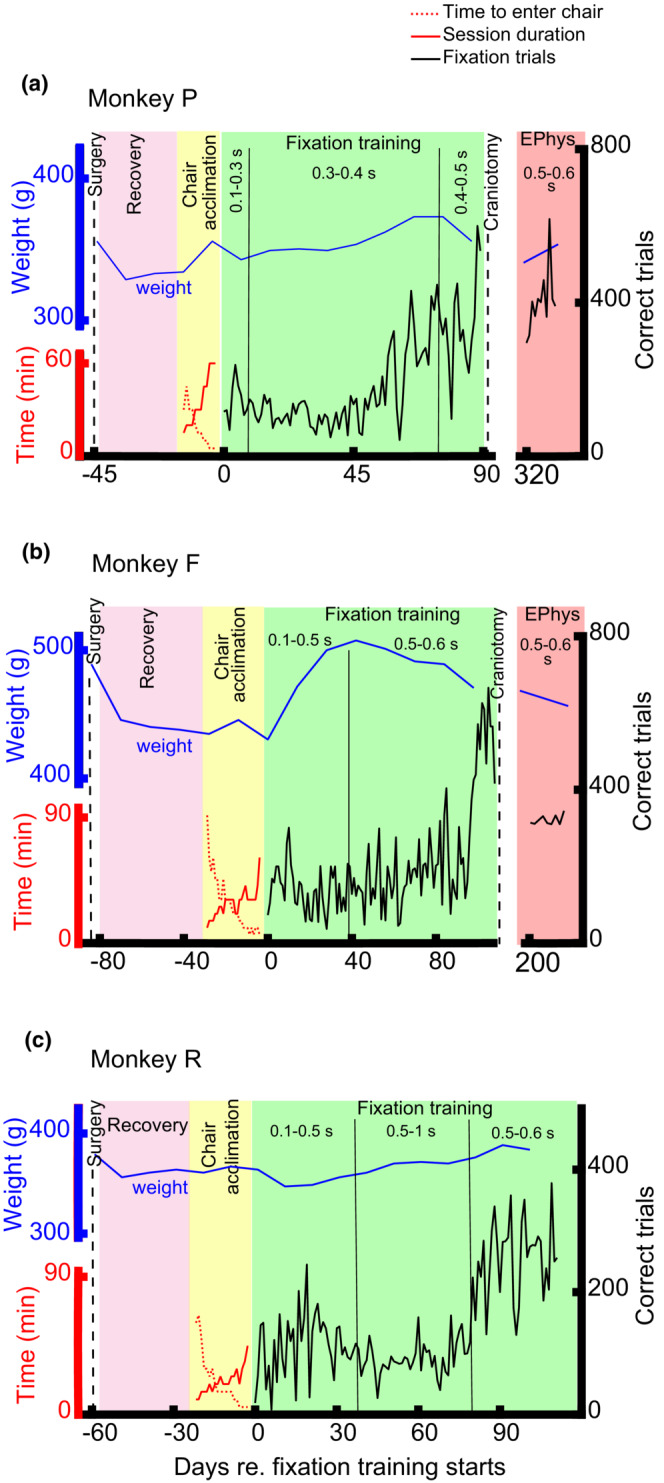
Training process for Monkey P, F, and R. *X*‐axis shows time, and 0 is the first day of fixation training. The vertical dash line depicts head‐post implant surgery, and the pink area shows the recovery period. The yellow area shows chair acclimation period. As training progressed, it took less time animals to being sit in their chair (red dotted line), and they stayed for a more extended time in their chair (solid red line). Black lines on the green and purple backgrounds show the number of completed trials in fixation training and electrophysiological recording sessions, respectively. As the black curves show, animals completed more trials by training. The blue line shows the animal's weight.

After calibrating the eye‐tracking system, we began fixation training by presenting a relatively large fixation point (3 degrees), and the animal was required to fixate within an 8 degrees window for 100 ms to receive a drop of juice, infant dried milk (guigoz 2; Nestle) or pineapple syrup. The reward was delivered using a syringe pump (ToosBioResearch). As the marmoset learned to fixate for longer durations, we gradually increased the required fixation time (up to 600 ms as we required for our experiment) and reduced the FP size to 0.5 degrees and the fixation window to 3 degrees (Figure 3).

### 2.5 Extracellular recording

Three days after craniotomy, neurophysiological recordings commenced. Single insulated tungsten microelectrodes (~0.5–2 MΩ; Thomas Recordings) or 16 channels linear array electrodes (Thomas Recordings) were lowered, at a posterior angle of 38 degrees, into the left SC through a stainless‐steel guide tube (22‐gauge needle) using an electronic two‐stage Microdrive (ToosBioResearch). The guide tube stopped underneath the dura and the electrode penetrated the brain. The neural signal was recorded at 20 kHz, and the eye tracker and the photodiode signals were recorded at 1 kHz for offline analysis (USB‐ME‐64 System; Multichannel Systems). Action potentials were detected by applying a threshold to the filtered (Butterworth band passed filter; 0.3–5 kHz) raw voltages and sorted post hoc for further analysis. After isolating units with large action potentials, we mapped the visual receptive field and motor field of neurons.

**FIGURE 4:**
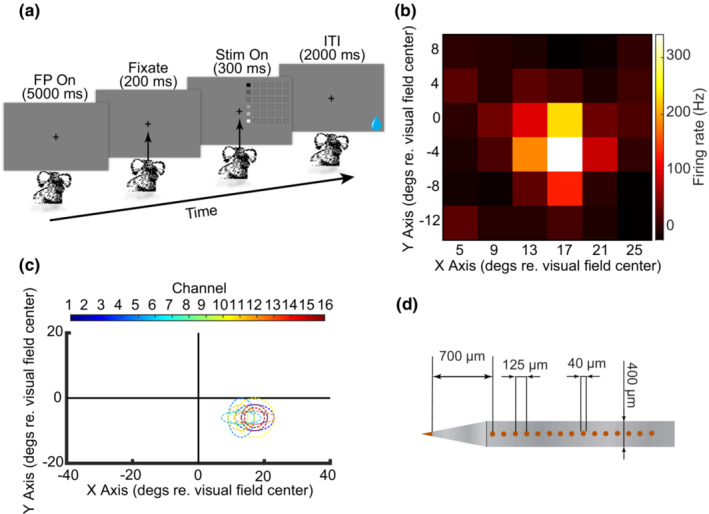
Visual receptive field mapping using reverse correlation. (a) Visual stimuli presented at 6 locations of one column of a checkerboard area for 50 ms each. (b) Visual response reverse correlated with the stimuli positions on the screen shown as a heat map. (c) Visual receptive field positions reconstructed for 16 channels (the color bar above the plot represents the color of each channel's RF) of a linear array electrode that are highly overlapped. (d) Depiction of the linear electrode array used in this experiment with dimensions and the distance between channels 1 to 16.

To map the visual receptive field of SC neurons with minimal numbers of trials, in each trial the animal fixated the FP and a white square patch (2 degrees) presented at six locations, 50 ms each, on a column of a 6 × 6 checkerboard area (size 22 × 22 degrees; Figure 4a). The stimulus patches were randomly presented at each of the six columns for at least five repetitions. Response latency was subtracted, and responses to the visual stimuli were correlated with the start time of each stimulus patch, captured by the photodiode signal, using formula 2:

Formula (2):

$$C\left(t^{\prime }\right)=\frac{1}{n}\times \frac{1000}{∆t}\times \sum_{t=1}^{T}S_{t}\left(V_{t-t^{\prime }}\right)$$

where *n* is the number of repetitions, Δ*t* is the stimulus presentation time (*T* − *t*), *t*' is the response latency of the neuron to the visual stimuli, *S* is the spike count, and *V* is the visual stimulus.

The results of reverse correlation were plotted as a 2D color‐coded map, showing RF location as a bright spot (Figure 4b).

**FIGURE 5:**
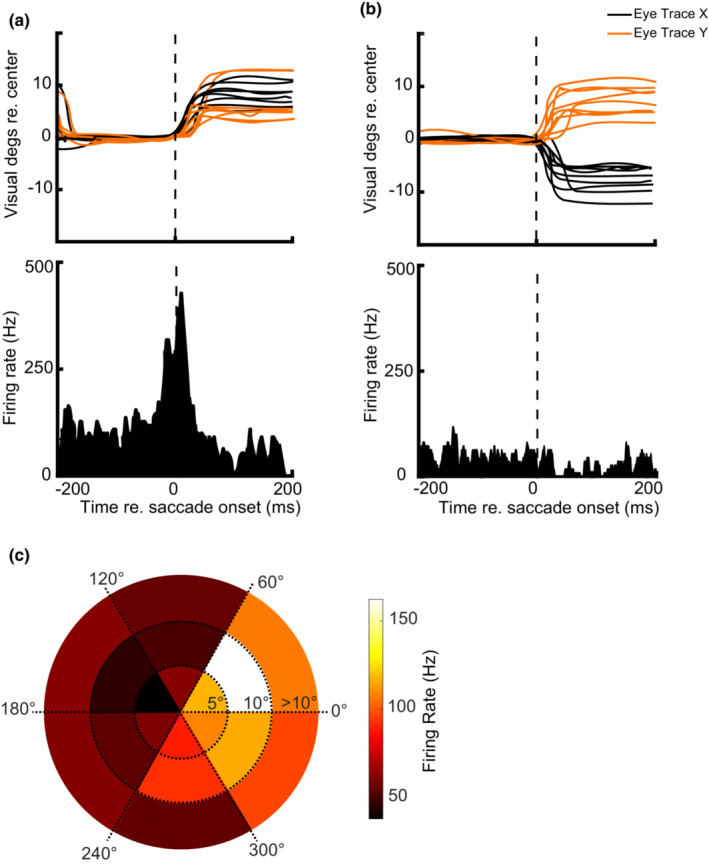
Motor field mapping using reverse correlation. (a) *X* (black) and *Y* (brown) eye trace for spontaneous saccades to the upper right quadrant of the visual field and PSTHs aligned to saccade onset. (b): *X* (black) and *Y* (brown) eye trace for spontaneous saccades to the upper left quadrant of the visual field and PSTHs aligned to saccade onset. The neuron was active around the time of saccades to the upper right hemifield but not to saccades to the upper left hemifield. (c) The heat map shows reverse‐correlation of spiking activity with saccades amplitude‐direction for one channel in a larger scale.

In addition to responding to visual stimuli, SC neurons fire a burst of action potentials around the time a saccade is launched into a specific location in the contralateral hemifield, their motor fields (Gandhi & Katnani, 2011). In macaque monkeys that perform more than a thousand trials in an experimental session, researchers run tens of trials in a memory‐guided saccade task to define the visual and motor field of the SC neurons under study. In marmosets, the number of trials performed in a session is more limited. We used the reverse correlation method to define the motor field of SC neurons while marmosets were freely viewing the screen without performing any task, so saving trials for the main task. First, we detected saccades and determined the saccade length and direction (Cloherty et al., 2020). The horizontal and vertical eye signals smoothed by a 20 ms moving averaging filter, and the angular velocity and acceleration calculated for eye traces. The velocity larger than 100 degrees/sec considered a potential saccade. Candidate saccades were further processed if the acceleration exceeded 5000°/s^2^. In a 100 ms window before peak velocity, the first time that the eye velocity exceeded 20°/s considered as the saccade onset. For each saccade, we determined the saccade length and direction. Spiking activity 50 ms before to 50 ms after the saccade onset correlated to the eye movement's length and direction. The correlation results were categorized into six groups based on saccades direction in which each group covered 60 degrees of the visual angle in a polar coordinate system. Based on their length, the saccadic eye movements were separated into short (<5 degrees), medium (5–10 degrees), and long (>10 degrees). Finally, the results were plotted as a polar heat map (Figure 5).

## 3 RESULTS

### 3.1 Chair training

Before training marmosets in the oculomotor tasks, the animals must be trained to sit comfortably and cooperatively in their chair with their head being restrained. We used a squeeze‐walled transfer box to guide the subjects into their chair. After a few acclimation sessions, the animals were immediately moved into the box to receive a palatable treat like mealworms, marshmallows, and acacia gum. Then, the box was positioned on an aluminum profile underneath the chair tube, with the box's circular opening contacting the lower edge of the chair tube (Figure 1b). Before moving the squeeze‐wall, the exit door was opened, so the animals were guided, not squeezed, to the chair tube. After few sessions, the animals entered chair tube before moving the squeeze wall. In the first training session, it took a long time for the marmosets to enter their chairs, 90, 30, and 60 min for monkeys F, P, and R, respectively (Figure 3; red dotted line). After few days of training, the animals immediately and voluntarily entered the squeeze‐walled transfer box. As the chair training progressed, the entire time needed for the animals to being head restrained decreased to less than 5 min after 25, 10, and 17 sessions of training for monkeys F, P, and R, respectively. The fact that the marmoset so swiftly and voluntarily entered the box, despite having full access to water and food in their home cages, suggests that they comfortably accepted the procedure, and it is very likely they considered it as an enrichment session. In the early training sessions, all three animals showed signs of discomfort after 10–15 min being sat in their chair, so, they were returned to their home cages immediately. As the acclimation progressed, they also sat comfortably in their chair for a longer duration (monkey F 60 min after 27 days of training, monkey P 60 min after 10 days of training, and monkey R 45 min after 20 days of training, Figure 3; red line). Overall, these observations show that chair acclimation of the marmoset can be done in 2–4 weeks, and it very likely causes less stress to the animal relative to the current handling methods. This squeeze‐walled chairing method in marmosets might be comparable to the collar‐free chairing method of the macaque monkey which is recommended to substitute the traditional pole‐collar method buy IACUCs in many institutions (McMillan et al., 2014; Ponce et al., 2016).

### 3.2 Eye calibration and fixation training

Both marmosets spontaneously fixated on highly salient flickering stimuli without being rewarded (Figure 2b). During a 10‐min period, marmosets P and F fixated 76 and 51 times, respectively. To exclude random fixations at locations other than flickering stimuli, we included only fixations of longer than 500 ms. In the Figure 2b, fixations during display of each stimulus are shown in different colors, and the fixations during presentation of each stimulus clustered around locations of the flickering stimuli. (Locations of flickering stimuli are shown as green circles). The centers of these five clusters were determined by fitting a Gaussian function to the data (Figure 2c). The Gaussian distribution excluded few random fixations at locations other than flickering stimuli. The standard deviation of these five clusters was 2.12 and 1.26 degrees for monkey P and F, respectively. Using the centers extracted from Gaussian fits, we set the gain and removed the offset for the *X* and *Y* axes. Once we performed this linear calibration, in the subsequent experimental sessions, we removed the offset by visually evaluating fixations. Average distance between stimulus locations and eye direction was less than 0.5 degree for most of experiments (69 out of 76 sessions: Figure 2d,e). This was expected as the animal head was fixed using four receptacles to the chair, and the location of chair and the eye tracker camera was unchanged across the study. Moreover, the offset and gain monitored and could be adjusted at the beginning of each experiment if necessary. Using this protocol, the eye‐tracking system for both untrained marmosets was easily calibrated in less than 15 minutes (10 min for eye signal recording and 5 min for subsequent analysis).

Fixation training in the head‐restrained marmosets began with a large FP, and the animals were required to fixate within an 8‐degree window for 100 ms to receive a reward. As the fixation training progressed, the marmosets held fixations for longer durations, and they completed higher number of fixations within a 3‐degree window (Figure 3). During the first few weeks of training, monkeys F and P completed about 150 fixations for 500–600 ms and 400–500 ms, respectively, and monkey R completed about 100 fixations for 500–1000 ms. They completed 500 (monkey F), 400 (monkey P), and 300 (monkey R) trials after 100 and 60, and 80 days of training, respectively. During neural recording, monkeys P and F performed around 300 and 400 trials per day, respectively. All trainings performed without restricting the animals access to food or water.

### 3.3 Receptive field mapping

After marmosets became proficient in fixating an FP for at least 600 ms for more than 300 trials per session, a craniotomy under general anesthesia was performed, and the neurophysiological experiments started. For each penetration, a tungsten microelectrode or a 16‐channel linear array electrode was lowered through a guide tube to the left superior colliculus. About 10 mm below the brain surface, we searched for conspicuous spikes and visual responses. After isolating high amplitude spiking activity, we mapped visual receptive fields as described above (Figure 4a). Neural responses to a visual stimulus presented at different locations computed by reverse correlation, and the result plotted as a 2D heat map (Figure 4b). We performed this analysis to define RF locations for all 16 recording channels, and as this was expected (because the electrode was positioned almost perpendicular to the SC) RF of most channels were highly overlapped (Figure 4c). The visual to motor response ratio (mean response to the visual stimulus presented at the cell's RFs/mean peri‐saccadic activity) decreased across 2500 micrometers depth of the SC. Superficial neurons responded more vigorously to visual stimuli, and deeper neurons had stronger motor‐related activity. This was similar to SC physiology in other vertebrate species like macaque monkeys (Massot et al., 2019), birds (Knudsen, 2011; Krauzlis et al., 2018), rodents (Basso & May, 2017), and reptiles (Basso et al., 2021; Stein & Gaither, 1981).

### 3.4 Motor field mapping

The SC neurons fire a burst of action potentials around the time of a saccadic eye movement with a specific direction and amplitude (re. retinal visual axis), an area called their motor field. Figure 5a,b represents eye traces and PSTHs for eight saccades with an amplitude of 9 ± 3 degrees and direction of 33 ± 11 degrees visual angle (upper right quadrant) and nine saccades with an amplitude of 9.5 ± 2.75 degrees and direction of 140 ± 14.3 degrees visual angle (upper left quadrant), respectively. The neuron fired vigorously around the time of saccades to the upper right quadrant while it was almost silent around the time of saccades to the upper left visual field quadrant. Although using reverse correlation to define RFs is not uncommon, we could not find any study using this method to define neuronal motor fields. To define the motor field of SC neurons, we correlated saccade direction and amplitude to the spiking activity of neurons. A colormap of eye movement‐spiking activity correlation is plotted for one channel in Figure 5c. Motor fields for neurons across all 16 channels were located at the upper right quadrant of the visual field, contralateral to the recording site, with considerable overlap (data not shown). Moreover, the motor field locations overlapped with visual receptive fields recorded at the same site.

The advantages of using the saccade‐spike correlation method are twofold: First, it is based on spontaneous saccade analysis, so it does not require a monkey to do trials thus saves trials for the main task. Moreover, it is easily doable on data collected from all channels of an array, and in contrast to the microstimulation technique, it does not require independent testing of every channel.

## 4 DISCUSSION

Unlike visual and oculomotor studies in macaque monkeys that dates back to the 1970s (Goldberg & Wurtz, 1972; Wurtz, 1969b) and enjoys the well‐established behavioral and electrophysiological techniques, neuronal recording and manipulation in behaving marmosets in oculomotor tasks has started recently. Researchers have attempted to characterize the cognitive capacity of the marmoset (Chen et al., 2021; Cloherty et al., 2020; Johnston et al., 2018; Mitchell et al., 2014), to establish new behavioral paradigms, to characterize the brain areas that are well characterized in the macaque monkeys (Ghahremani et al., 2019; Selvanayagam et al., 2019; Solomon & Rosa, 2014), and to improve general experimental tools and protocols for more efficient neural recording and manipulation in behaving marmosets (Ebina et al., 2019; Johnston et al., 2018; Macdougall et al., 2016; Sedaghat‐Nejad et al., 2019). In this report, we improved protocols for handling and chairing, eye calibration, and receptive and motor field mapping that is fundamental to neurophysiological and behavioral studies under head‐restraint in the marmoset.

Acclimating to sit calmly in a chair under head‐restrained condition is fundamental to training the marmoset in visual and oculomotor tasks. Acclimation is a very time‐consuming procedure across reports, and investigators usually have not described this time‐consuming procedure in enough detail (Several weeks in Wang's laboratory, see Lu et al., 2001; 3–4 months in Miller's laboratory, see Nummela et al., 2017 and Mitchell et al., 2014; more than 200 days in Shadmehr's laboratory, see Sedaghat‐Nejad et al., 2019; 6–8 weeks in Everling's laboratory, see Johnston et al., 2018). Investigators usually use a plexiglass tube to transfer marmosets from a transfer box to a chair, a protocol that requires handling and touching the animals (Lu et al., 2001; Remington et al., 2012). The marmoset is a small prey species and dislikes being touched and grabbed by the experimenter's hand, so it takes a long time for them to get used to this stressful procedure. We successfully tackled this challenge by designing a squeeze‐walled plexiglass transfer box that guided marmosets to the chair tube without requiring the experimenter to touch them directly. The squeeze‐wall was not used to reduce the space to immobilize the marmoset (as it is used to chair or inject macaque monkeys), instead the wall was squeezed only when the exit door was open; thus, it simply guided the subject to the next compartment, that is, chair tube. Using the squeeze‐walled box, chairing marmosets under head‐restrained conditions was possible in a few days, and in less than 4 weeks, all three subjects were fully acclimatized and were ready for eye calibration and task training. An even more important advantage of this protocol is that it is less dependent on experimenters' skills for interacting with animals, resulting in more consistency over experimental days, experimenters, and animals. The described improvement in chair training can help researchers to significantly reduce required time and resources to prepare an animal for behavioral and neurophysiological experiments.

More importantly, the stress level of the animals during a procedure is a concern of primate researchers and the overseeing committees. One major advantage of this new method is to address this concern. Our new volunteer chairing of the marmoset is comparable to use of volunteer chairing of macaque monkeys that has replaced traditional pole‐collar method in many primate laboratories (McMillan et al., 2014; Ponce et al., 2016). Since this method helps to avoid contacting the animal, relative to other handling methods, it most likely is less stressful to the animal. Although we did not measure stress indicators (like blood cortisol level), the animals behavior suggests that this method is less stressful to them than common methods: First, as the training progressed, the time took the animal to voluntarily enter and sit in the chair decreased. After a few sessions of training, the entire process (animal entering the squeeze‐wall box, transfer of the box to the laboratory, positioning the box under the chair, the animal entering the chair, stabilizing the animals head using four pins on the chair) took less than 10 min. Second, although the animals had full access to water and food, they voluntarily almost immediately entered the box (Figure 3, red dotted curves). After head stabilization, the animals stayed in the chair without showing any sign of discomfort. It is very likely that the animals thought of the process as enrichment rather than work. Overall, less stressful procedure might be the reason of shorter acclimatization process in this study relative to the other ones.

To calibrate the eye‐tracking system, the subject has to fixate its gaze on specified locations on the screen for a short period, which is not straightforward in an untrained marmoset. We recorded the eye traces of the freely viewing subject during 2 min in which a highly salient flickering‐colored stimulus displayed on specified locations on the screen. This mesmerizing stimulus regimen frequently attracted a naive marmoset's gaze for a relatively long duration. This protocol did not depend on the real‐time recognition of fixations on the stimulus by the experimenter; it was quick (10 min free viewing), and it did not require the animal to be engaged in a task. Therefore, if a re‐calibration becomes necessary, it is easily feasible at the beginning of an experimental session. Some investigators used a salient stimulus like a marmoset face (Ghahremani et al., 2019; Mitchell et al., 2014; Nummela et al., 2017; Selvanayagam et al., 2019), or colored spots or moving pictures (Spadacenta et al., 2019) to make the subject fixate for a short period and manually captured fixations (e.g. by a key press), a process sometimes is frustrating to do for a naïve marmoset (Mitchell et al., 2014). Mitchell et al. (2014) used marmoset faces as stimuli, and calibrated the eye tracker's gain and offset offline, as we did in this study. However, since the flickering stimuli are more salient than face pictures, the untrained marmosets spontaneously and frequently fixated the stimuli; thus, we were able to perform it in free viewing marmosets. Moreover, we presented stimuli for 10 min and registered tens of long fixations for each location so that fitting a gaussian function was possible, yielding a quantitative and accurate fixation center. Recently Yates et al. (2021) used the Dual‐Purkenje Image eye‐tracking system in the marmoset to map foveal receptive field in the V1. Although the technique is more precise, calibration depends on neural recording, so only possible in experiments with neural recording (Yates et al., 2021). Jendritza et al. (2021) used a large stimulus for initial calibration. Utilizing the rudimentary initial calibration, in the main calibration session, they presented stimuli at specific locations and they rewarded the animal for fixating for at least 150–300 ms; then, they calculated the gain and offset of their tracking system by offline processing of the fixations. Although it is accurate, their initial calibration requires recognition of gazes by the experimenter, and their main calibration requires the cooperation of an untrained animal to perform trials in an independent session. Collecting hundreds of fixations for all calibration points requires a subject to perform hundreds of trials which would take an entire good experimental session. Overall, calibration in our protocol is quick, accurate, easily doable in a untrained free viewing animal.

In the fixation task (for 600 ms), the animals performed about 300 trials after 60 days of training and about 500 trials after 90 days of training without any food or water restriction. The animals were rewarded 0.02–0.03 ml juice per trial. In other studies, with comparable reward regimes and without fluid restriction, the marmosets performed a comparable number of trials (Mitchell et al., 2014; Spadacenta et al., 2019). In some studies, animals received larger rewards (0.07 ml per trial) and performed fewer trials (Johnston et al., 2018). In some other studies, the marmosets performed even a smaller number of trials per session, maybe due to task complexity (Mitchell et al., 2015). By restricting marmosets' food and fluid access and providing a minimal amount of reward per trial during experiments, Shadmehr's laboratory managed to run more than 1000 trials per session (Sedaghat‐Nejad et al., 2019). We think that with improvements in chair acclimatization and eye calibration protocols in this report, in combination with food managements reported by Shadmehr's laboratory, training marmosets can be done in a reasonably short time period with enough trials per experimental session.

Since the 1970s that Robert Wurtz and his colleagues pioneered neuronal recording in behaving macaque monkeys, extensive neuronal recording and manipulation established the SC as an essential structure for eye movement, spatial attention, and decision‐making (Basso & May, 2017; Krauzlis et al., 2013; Wurtz, 1969a; Wurtz & Goldberg, 1971). Past studies using in vivo neuronal recording and microstimulation characterized two main cortical oculomotor areas, namely the frontal eye field and posterior parietal cortices in awake marmosets (Ghahremani et al., 2019; Ma et al., 2020; Selvanayagam et al., 2019). This study is the first report of the electrophysiological recording of the superior colliculus in awake marmosets (Tailby et al., 2012). Additionally, we adopted techniques for quick mapping of visual receptive and motor fields in behaving marmosets in oculomotor tasks. We adopted the reverse correlation method to characterize visual receptive fields of SC neurons, a technique that is quick and can be done with few trials and saves trials for the main task. Recently, Yates et al. (2021) used reverse correlation to estimate the receptive field of V1 neurons in freely viewing marmosets (Yates et al., 2021). Although their method has an extraordinary high resolution to characterize foveal V1 neuron's receptive fields in freely viewing animal, their methods require more sophisticated hardware and software. However, we believe that a variation of their protocol, with a simpler hardware and software, can be adopted for estimating receptive fields in freely viewing marmosets.

In this study, we correlated saccade direction and amplitude to the spiking activity of neurons to define the motor field of SC neurons. Past studies used VGS, MGS, or brain stimulation to define motor field of cortical and sub‐cortical structures (Massot et al., 2019). The advantages of using the saccade‐spike correlation method are twofold: First, it is based on spontaneous saccades of a free viewing marmoset, so it does not require the subject to perform trials, thus it saves trials for the main task. More importantly, it could be used to map motor filed of neurons recorded from all channels of a linear electrode array recorded simultaneously, and it does not require independent testing of each channel, as is needed for motor mapping using the microstimulation technique.

Since we penetrated the SC almost perpendicularly (38 degrees posteriorly angled) with linear arrays, receptive fields of units at different depths (i. e., layers) were highly overlapped. In addition to collecting more data, this method allows investigating information flow and processing across various SC layers. Consistent with SC in macaque monkeys and other vertebrates, superficial layers responded stronger to visual stimuli, and deeper layers were active around the time of the eye movements. The reverse‐correlation method for visual receptive field and motor field characterization was accurate, quick, and did not require marmosets to perform many trials in a complex task.

This report introduces a more efficient and less stressful method for chair training, a refined eye calibration protocol in freely viewing marmosets, and motor field mapping of oculomotor neurons in freely viewing subjects. Moreover, combination of these fundamental procedures in a single study provides an efficient and refined package for electrophysiological experiments in oculomotor tasks. Finally, this is the first report of use of linear electrode arrays for recording neuronal responses across all SC layers in behaving marmosets.

## 5 CONCLUSION

The marmoset's brain and behavioral similarity to humans and its amenability to biotechnological techniques make it a promising experimental model to study cognition's cellular and synaptic basis. We refined protocols for chair acclimation, eye calibration, and motor field mapping of SC neurons that shortened the acclimation period, most likely reduced stress to the subjects, and allowed more efficient eye calibration and motor field mapping in freely viewing marmosets. We established extracellular neurophysiology using linear electrode arrays across SC layers in behaving marmosets.

## AUTHOR CONTRIBUTIONs

Ali Asadollahi and Seyed Javad Saghravanian designed the experiments, formulated the analysis, and wrote the manuscript. Seyed Javad Saghravanian collected the data. Ali Asadollahi supervised the project.

## FUNDING INFORMATION

The project was supported by Ferdowsi University of Mashhad (grant number of 3/46867) and also, the Iranian Cognitive Sciences and Technologies Council (grant numbers of 553 and 1275).

## CONFLICT OF INTEREST STATEMENT

The authors have no conflicts of interest.

## ETHICS STATEMENT

All procedures were performed following the protpcol approved by the Etics Comittee for Animal Care and Use of the Ferdowsi University of Mashhad witht the code number of IR.UM.REC.1399.145.
